# Comparative Study of 27-Gauge versus 25-Gauge Vitrectomy for the Treatment of Primary Rhegmatogenous Retinal Detachment

**DOI:** 10.1155/2017/6384985

**Published:** 2017-03-04

**Authors:** Stanislao Rizzo, Silvio Polizzi, Francesco Barca, Tomaso Caporossi, Gianni Virgili

**Affiliations:** ^1^Ophthalmology Department, University of Florence, A.O.U.C., Largo Brambilla 3, 50134 Florence, Italy; ^2^Pediatric Ophthalmology Unit, A. Meyer Children's Hospital, Florence, Italy

## Abstract

*Purpose*. To compare the vitrectomy time, clinical outcomes, and complications between 27-gauge (27-G) and 25-gauge (25-G) vitrectomy in patients with primary rhegmatogenous retinal detachment (PRRD). *Methods*. Prospective, nonrandomized, comparative, interventional study. Forty consecutive patients with PRRD were recruited. Twenty patients underwent the 27-gauge procedure and twenty patients had the 25-gauge procedure. The main outcome measure of the study was the actual vitrectomy time. *Results*. The mean duration of vitreous removal was 23.2 min (SD 6.5) with 27-G vitrectomy and 19.6 min (SD 7.3) with 25-G vitrectomy, resulting in a difference of 3.6 min (95% confidence interval (95%CI): −8.0 to 0.8 mins, *p* = 0.11). Mean logMAR visual acuity improved from 1.70 ± 1.18 preoperatively to 0.12 ± 0.14 at final postoperative visit (*p* < 0.001) in the 27-G group and from 1.52 ± 1.15 preoperatively to 0.22 ± 0.30 at final postoperative visit (*p* < 0.001) in the 25-G group. The anatomical success rate after a single operation was 90.0% and 85.0% in the 27-G and in the 25-G groups (*p* = 0.63), respectively. Intraoperative iatrogenic retinal breaks (IRBs) occurred in 2 eyes in the 27-G group and 1 eye in the 25-G group. *Conclusions*. Twenty-seven-gauge vitrectomy may be a safe and effective surgery for the treatment of PRRD.

## 1. Introduction

The introduction of pars plana vitrectomy (PPV) in the early 1970s by Machemer et al. [1] represented a milestone in ophthalmic progress because for the first time, it allowed for the removal of the vitreous through a closed system rather than through an open-sky technique. Since that moment, the evolution of vitrectomy instrumentation has been driven by the desire for smaller instruments and greater functionality. One of the main aims has been to make smaller and smaller wounds to reduce the surgical trauma, recovery times, and postoperative complications. Over the past several years, recent innovations, such as the advent of powerful light sources, stronger instruments, and high-performance vitrectomy machines, have led to the development of a 27-gauge (27-G) transconjunctival sutureless vitrectomy (TSV) system [2]. The feasibility of this new microincision vitrectomy surgery (MIVS) has been recently demonstrated for various vitreoretinal diseases [2–10], including rhegmatogenous retinal detachment (RRD) [4–6, 8–10]. However, to our knowledge, to date, there is only one comparative study between 27-G and 25-G vitrectomy systems for RRD [10]. The purpose of this study was to compare the surgical time, clinical outcomes, and complications between 27-G and 25-G vitrectomy surgery in patients with primary rhegmatogenous retinal detachment (PRRD).

## 2. Materials and Methods

A prospective, nonrandomized, comparative study was performed on 40 consecutive patients with PRRD undergoing 27- or 25-gauge TSV. Twenty patients (50%) were treated with the 27-gauge procedure and twenty (50%) with the 25-gauge procedure. All surgeries were carried out by two surgeons at a single center between July 2015 and October 2015. Each surgeon operated on a similar number of patients in both groups. Inclusion criteria were PRRDs with one or more retinal breaks and the ability to give informed consent. Exclusion criteria included a follow-up period of less than 6 months, patients judged to be incapable of postoperative posturing, a history of any previous vitreoretinal surgical procedures or penetrating ocular trauma, proliferative vitreoretinopathy (PVR) of grade C or greater, and significant ocular comorbidities such as uveitis, uncontrolled glaucoma, and severe or proliferative diabetic retinopathy. Preoperative evaluation consisted of a complete medical, surgical, and ophthalmic history followed by a thorough ophthalmic examination. Preoperative data included age, sex, the eye's axis length, the best-corrected visual acuity (BCVA), intraocular pressure (IOP), lens status, extent of retinal detachment, and location of breaks. At the end of every surgery, the vitrectomy time and intraoperative complications were recorded. Postoperative examination was carried out on 1 day; 1 week; and 1, 2, 3, and 6 months. Postoperative data collected included the retinal status, postoperative complications, and BCVA at 1, 2, 3, and 6 months. Intraocular pressure (IOP) was measured using Goldmann applanation tonometry, and severe postoperative hypotony and hypertony were, respectively, defined as IOP < 6 mmHg and IOP > 30 mmHg. The main outcome measure of the study was the actual vitrectomy time. It was defined as the time required for the complete removal of the vitreous or rather the time period when the cutter was activated for removing the vitreous. To evaluate it, we have recorded the cutting time reported by the instrument. The secondary outcome measures were primary anatomical success rate, postoperative BCVA, and intra- and postoperative complications. BCVA was recorded as a Snellen visual acuity and converted to logarithm of minimal angle of resolution (logMAR) units for statistical analysis. Counting finger (CF) vision was defined as 2.0 logMAR and hand movements (HM) were defined as 3.0 logMAR. For visual outcome comparisons, we excluded patients with amblyopia or retinal redetachment. A primary anatomical success was defined as a complete reattachment of the retina following the initial surgery, when all the gases in the eye had disappeared or silicone oil was removed. The study followed the tenets of the Declaration of Helsinki and was approved by the institution's review board. Written informed consent was obtained from all patients.

### 2.1. Surgical Technique

All surgeries were performed under a retrobulbar block, using the Constellation vitrectomy system (Alcon, Fort Worth, TX, USA). For this study, the machine was set with an initial aspiration of 0 mmHg moving linearly to 650 mmHg when the foot pedal is fully depressed, maintaining a fixed cut rate of 7500 cuts per minute (cpm) in both vitrectomy systems (27 + and 25 + Total Plus Pak). During surgery, IOP was controlled to 25 mmHg. For posterior visualization, Resight 700 (Carl Zeiss Meditec AG, Oberkochen, Germany) was used. Both the 27- and 25-gauge procedures were performed using a four-port pars plana technique (the fourth port was for a 25-gauge chandelier illuminator). Before starting the surgery, the eyelid and periorbital skin and the ocular surface were prepared with 5% povidone-iodine. After the conjunctiva was displaced slightly, the trocars were placed through the conjunctiva and the sclera 3.5 mm from the limbus. The sclerotomy was created using the trocar cannula with a biplanar entry, tangential to the sclera at first, and then perpendicularly thereafter to create a self-sealing incision, as much as was possible. Phacoemulsification was performed in all phakic eyes to help in the complete removal of the anteroperipheral vitreous. Complete removal of the vitreous gel was performed. Triamcinolone acetonide was routinely injected to facilitate visualization of the vitreous base which was meticulously shaved circumferentially. Scleral indentation was performed with a metal scleral depressor. Any tears or suspicious retinal lesions were treated with endolaser photocoagulation or transscleral cryopexy. Intraoperative use of perfluorocarbon liquids (PFCL) was at the discretion of the operating surgeon. After air-fluid exchange, 20% sulfur hexafluoride (SF6) gas, 12% perfluoropropane (C3F8) gas, or 1000 centistoke silicone oil was used as the tamponade. Silicone oil was given at the discretion of the operating surgeon, or to patients who had to take an aeroplane or who needed to have early visual rehabilitation. After vitrectomy, the microcannulas were removed and a gentle massage of the sclerotomy with a cotton-tipped applicator was performed to avoid leakage; otherwise, bipolar diathermy was performed. If any site showed persistent leakage, 8-0 vicryl sutures were placed in the wound and the overlying conjunctiva. At the completion of the surgery, peribulbar injections of antibiotics and dexamethasone were given. Patients were asked to pose for 7 days, either face down or on one side depending on break position. In both groups, patients received eye drops containing antibiotics and dexamethasone with tapered frequency during the 4 weeks after surgery. During the follow-up period, antiglaucoma eye drops, such as beta-blockers, carbonic anhydrase inhibitors, or prostaglandin analogues, were prescribed when IOP was higher than 24 mmHg. The patients who received silicone oil tamponade underwent a second surgical procedure to remove the oil within 4 months of the initial surgery.

### 2.2. Statistical Analysis

A linear mixed model was used to compare the continuous measures in the two groups. Anatomic success at 6 months as a dichotomous measure was compared using a chi-square test. A *p* value of <0.05 was defined as statistically significant.

## 3. Results

### 3.1. Preoperative Characteristics

The mean age of the patients was 64.7 ± 9.7 years (range: 46–78 years) and 62.4 ± 9.8 years (range: 48–83 years) in the 27-G and 25-G groups, respectively. There were 15 men (75%) and 5 women (25%) in the 27-G group and 14 men (70%) and 6 women (30%) in the 25-G group. Mean duration of visual loss was 6.1 ± 5.9 days (range: 1–20 days) in the 27-G group and 8.15 ± 8.77 days (range: 1–30 days) in the 25-G group. Baseline logMAR visual acuity (mean ± SD) was 1.70 ± 1.18 (range: 3.0 to 0.1) in the 27-G group and 1.52 ± 1.15 (range: 3.0 to 0.1) in the 25-G group. Clinical data of the patients are given in Table 1. There were no statistically significant differences in the patients' preoperative characteristics between the 27-G and 25-G groups.

### 3.2. Surgical Time and Results

All phakic eyes in each group had simultaneous phacoemulsification with intraocular lens implantation to help in the complete removal of the anteroperipheral vitreous. No complications occurred related to phacoemulsification such as posterior capsule rupture or zonular dialysis. The mean duration of vitreous removal was 23.2 min (SD 6.5) with 27-G vitrectomy and 19.6 min (SD 7.3) with 25-G vitrectomy, resulting in a difference of 3.6 min (95% confidence interval (95%CI): −8.0 to 0.8 mins, *p* = 0.11). PFCL was given in 18 eyes (90%) in each group. In the 27-G group, 14 eyes (70%) received endolaser and 6 eyes (30%) external cryoapplication, while in the 25-G group, 15 eyes (75%) received endolaser and 5 eyes (25%) external cryoapplication. In the 27-G group, 10 eyes (50%) had SF6 gas tamponade, 8 eyes (40%) had C3F8 gas tamponade, and 2 eyes (10%) were treated with silicone oil. The oil was given through the port for the chandelier illuminator. In the 25-G group, 7 eyes (35%) had SF6 gas tamponade, 7 eyes (35%) had C3F8 gas tamponade, and 6 eyes (30%) had silicone oil tamponade. After removal of the microcannulas, one sclerotomy site in 2 eyes (3.33% of sclerotomies) was sutured because of leakage in the 27-G group, while in the 25-G group, an average of two sclerotomy sites in 4 different eyes (20% of eyes, 13.3% of sclerotomies) were sutured for wound closure. In this calculation, we had excluded sclerotomy sites for the chandelier light source because we used a 25-G chandelier in the 27-G group, too. Silicone oil was removed after an average of 86.5 days (range: 78–95 days) after the first surgery in the 27-G group and after 66.6 days (range: 51–89 days) in the 25-G group. In both groups, this surgery was performed using the 25-G TSV system.

### 3.3. Anatomical Results

The primary anatomical success rate after a single operation was 90.0% and 85.0% in the 27-G and in the 25-G groups (*p* = 0.63), respectively. In the 27-G group, 2 eyes developed a retinal redetachment within 1 month of the initial surgery. In the 25-G group, 2 cases detached within 1 month of primary vitrectomy, while 1 case of redetachment occurred within 2 months of surgery. The redetachments were due to proliferative vitreoretinopathy (PVR) in 3 eyes (1 eye in the 27-G group and 2 in the 25-G group), a new retinal break in 1 eye (in the 25-G group), and an opening of an original retinal break in 1 eye (in the 27-G group). All these eyes were reoperated using 25-G instruments. Two cases of PVR were treated with peeling and gas as the tamponade agent, while 1 case required membrane peeling, a relaxing retinotomy, and long-term tamponade with 5700 silicone oil. The eye with the new break received gas as the tamponade agent, while the one with the opening of an original break required a third surgical procedure in which silicone oil tamponade was used. The final attachment rate was 100% in both groups.

### 3.4. Changes of Visual Acuity

Baseline and final visual acuity were 1.70 ± 1.18 and 0.12 ± 0.14 and 1.52 ± 1.15 and 0.22 ± 0.30 in the 27-G and 25-G groups, respectively (*p* < 0.001 for each comparison). However, visual recovery could not be assessed in 5 patients undergoing reintervention for redetachment, since the use of intraocular gas, and in general, the postsurgical condition, prevented us from measuring potential visual functioning. Therefore, the change of postoperative visual acuity was compared in the two groups of 18 and 17 patients with primary anatomical success and is given in Table 2. Postoperative BCVA increased significantly in both groups between 1 and 6 months postoperatively (*p* < 0.001) for all comparisons. The mean difference between 27-G and 25-G vitrectomy was −0.095 logMAR (95%CI: −0.231 to 0.042 logMAR), favouring the 27-G vitrectomy, which was not significant (*p* = 0.174).

### 3.5. Intraoperative Complications

Iatrogenic retinal breaks (IRBs) occurred in 2 eyes (10%) in the 27-G group and 1 eye (5%) in the 25-G group during the vitreous base shaving. Intraoperative laser photocoagulation was carried out around the retinal breaks, and no IRB resulted in postoperative rhegmatogenous retinal detachment. In the 27-G group, we also experienced one case (5%) of choroidal detachment by infusion cannula slippage into the suprachoroidal space because of a sudden movement of the patient during scleral depression. The infusion line was disconnected from the partially disinserted cannula and was reconnected to another fully inserted cannula. The surgery then proceeded without complications.

### 3.6. Postoperative Complications

Severe hypertension (IOP > 30 mmHg) was detected in 1 eye (5%) in the 27-G group at 1 month postoperatively and in 2 eyes (10%) in the 25-G at 1 week postoperatively. All the eyes with an elevated IOP were treated with antihypertensive eye drops, and the IOPs returned to normal levels without glaucoma surgery. No other postoperative complications, such as severe hypotony (IOP < 6 mmHg), intraocular bleeding, choroidal detachment, or endophthalmitis, were noted in the follow-up period in either group.

## 4. Discussions

In this study, we have compared the actual vitrectomy time, clinical outcomes, and complications between 27-G and 25-G vitrectomy surgery in patients with PRRD.

The actual vitrectomy time was slightly longer in the 27-G group compared to the 25-G group (23.2 ± 6.5 versus 19.6 ± 7.3 min, resp.). The difference that was found between the 2 groups was attributed to the different internal diameters of the vitrectomy probe of the two vitrectomy systems used. Indeed, it is known that aspiration and flow rate are regulated by Poiseuille's law, which states that the velocity of the flow of a fluid through a tube is directly proportional to the pressure difference and to the fourth power of the radius of the tube and inversely proportional to the length of the tube and to the coefficient of viscosity, so that the 27-G system had a reduction of flow and consequently, a slightly longer time for the removal of the vitreous as compared to those of the 25-G. In a recent comparative study between 27-G and 25-G microincision vitrectomy for epiretinal membrane (ERM), Mitsui et al. [7] also reported a mean vitrectomy time longer in the 27-G group than in the 25-G group (9.9 ± 3.5 versus 6.2 ± 2.7 min, resp.). However, a comparison between the two studies is difficult as they differ for the vitreoretinal diseases treated and surgical parameters used (in the 27-G, vacuum of 0–600 mmHg and cut rate of 1000–2500 cpm). Indeed, flow rate of viscous materials, such as the vitreous humor, is influenced by the cut rates. High cut rates result in smaller vitreous pieces that are more easily aspirated through the probe for a reduced resistance to flow; thus, vitreous flow rates increased with the increasing cut rate [11].

In both groups, the anatomical success rate was similar to the one described in earlier reports which reported an anatomic success rate with a single procedure ranging from 74% to 95% using 25-G PPV [10, 12–16]. Therefore, although definitive comparisons between studies are usually difficult, as they differ in many parameters, our results showed that 27-G TSV was as effective as 25-G in reattaching the retina after initial surgery.

Postoperative BCVA increased significantly in both groups between 1 and 6 months postoperatively. Studies using 25-G PPV to repair pseudophakic RDs have reported postoperative visual acuities of 20/40 or better in 50–53% of eyes [12–14]. In two of these studies [13, 14], the mean duration of visual loss was approximately 15 days, preoperative VA was 20/50 or less in 82–83% of the eyes, and a macular detachment was found in 77–78% of the eyes. In the present study, final visual acuity was 20/40 or better in 16 eyes (80%) in the 27-G group and in 15 eyes (75%) in the 25-G group, respectively. This can be explained in part by the shorter mean duration of visual loss (approximately, 6 days in the 27-G group and 8 in the 25-G group), because preoperative VA was 20/50 or less in 16 eyes (80%) in the 27-G group and 17 eyes (85%) in the 25-G group, and a macular detachment was found in 75% in the 27-G group and 85% in the 25-G group of eyes. In the study by Horozoglu et al. [12], the mean duration of macular detachment was approximately 6 days, but preoperative VA was 20/50 or less in 100% of the eyes.

Intra- and postoperative complications were similar in both 27-G and 25-G groups. No eyes required conversion to larger-gauge instrumentation during surgery. The 27-G instruments were found to be of sufficient strength to perform all surgical maneuvers in all eyes by both surgeons.

## 5. Conclusion

There are limitations to our study, including the small number and a nonrandomization of the patients. Nevertheless, in our series, 27-G vitrectomy seems to be as safe and effective as 25-G vitrectomy in PRRD surgery. A randomized, controlled trial with a larger number of patients is needed to confirm the results obtained in this study.

## Figures and Tables

**Table 1:** Clinical data of patients.

	27-G group	25-G group
Axial length (mm)	25.07	25.33
Lens status (phakic), number (%)	6 (30%)	9 (45%)
Lens status (pseudophakic), number (%)	14 (70%)	11 (55%)
Clock hours of retinal detachment, mean	6.35	7.1
Number of retinal breaks/holes, mean	3.2	2.85
Giant tears, number (%)	1 (5%)	3 (15%)
Macula-off, number (%)	15 (75%)	17 (85%)
PVR, number (%)	0 (0%)	1 (5%) (PVR of grade B)

PVR: proliferative vitreoretinopathy.

**Table 2:** Changes of visual acuity in patients with primary anatomical success.

	27-G group	25-G group
	LogMAR	LogMAR
Baseline visual acuity	1.65	1.31
Postoperative 1 month	0.23	0.36
Postoperative 2 months	0.15	0.25
Postoperative 3 months	0.13	0.22
Final visual acuity	0.09	0.16

## References

[1] Machemer R., Buettner H., Norton E. W., Parel J. M. (1971). Vitrectomy: a pars plana approach. *Transactions of the American Academy Ophthalmology and Otolaryngology*.

[2] Oshima Y., Wakabayashi T., Sato T., Ohji M., Tano Y. (2010). A 27-gauge instrument system for transconjunctival sutureless microincision vitrectomy surgery. *Ophthalmology*.

[3] Oshima Y., Wakabayashi T., Ohguro N., Nishida K. (2011). A 27-gauge sharp-tip short-shaft pneumatic vitreous cutter for transconjunctival sutureless vitreous biopsy. *Retina*.

[4] Romano M. R., Scotti F., Vinciguerra P. (2015). 27-gauge vitrectomy for primary rhegmatogenous retinal detachment: is it feasible?. *Annals of the Academy of Medicine, Singapore*.

[5] Rizzo S., Barca F., Caporossi T., Mariotti C. (2015). Twenty-seven-gauge vitrectomy for various vitreoretinal diseases. *Retina*.

[6] Khan M. A., Shahlaee A., Toussaint B. (2016). Outcomes of 27 gauge microincision vitrectomy surgery for posterior segment disease. *American Journal of Ophthalmology*.

[7] Mitsui K., Kogo J., Takeda H. (2016). Comparative study of 27-gauge vs 25-gauge vitrectomy for epiretinal membrane. *Eye (London, England)*.

[8] Toygar O., Mi C. W., Miller D. M., Riemann C. D. (2016). Outcomes of transconjunctival sutureless 27-gauge vitrectomy with silicone oil infusion. *Graefe's Archive for Clinical and Experimental Ophthalmology*.

[9] Pavlidis M., Körber N., Höhn F. (2016). Surgical and functional results of 27-gauge vitrectomy combined with coaxial 1.8 mm microincision cataract surgery: a consecutive case series. *Retina*.

[10] Romano M. R., Cennamo G., Ferrara M., Cennamo M., Cennamo G. (2016). Twenty-seven-gauge versus 25-gauge vitrectomy for primary rhegmatogenous retinal detachment. *Retina*.

[11] Abulon D. J. (2015). Vitreous flow rates through dual pneumatic cutters: effects of duty cycle and cut rate. *Clinical Ophthalmology*.

[12] Horozoglu F., Yanyali A., Celik E., Aytug B., Nohutcu A. F. (2007). Primary 25-gauge transconjunctival sutureless vitrectomy in pseudophakic retinal detachment. *Indian Journal of Ophthalmology*.

[13] Acar N., Kapran Z., Altan T., Unver Y. B., Yurtsever S., Kucuksumer Y. (2008). Primary 25-gauge sutureless vitrectomy with oblique sclerotomies in pseudophakic retinal detachment. *Retina*.

[14] Kapran Z., Acar N., Altan T., Unver Y. B., Yurttaser S. (2009). 25-gauge sutureless vitrectomy with oblique sclerotomies for the management of retinal detachment in pseudophakic and phakic eyes. *European Journal of Ophthalmology*.

[15] Lai M. M., Ruby A. J., Sarrafizadeh R. (2008). Repair of primary rhegmatogenous retinal detachment using 25-gauge transconjunctival sutureless vitrectomy. *Retina*.

[16] Kunikata H., Nishida K. (2010). Visual outcome and complications of 25-gauge vitrectomy for rhegmatogenous retinal detachment; 84 consecutive cases. *Eye (London, England)*.

